# A Compact 380 GHz Zero-Bias Schottky Diode Detector for High-Sensitivity Radiometer Applications

**DOI:** 10.3390/mi17030352

**Published:** 2026-03-13

**Authors:** Huilin Tang, Yongsheng Deng, Dehai Zhang

**Affiliations:** 1 The CAS Key Laboratory of Microwave Remote Sensing, National Space Science Center, Chinese Academy of Sciences, Beijing 100190, China; tanghuilin23@mails.ucas.ac.cn (H.T.); zhangdehai@mirslab.cn (D.Z.); 2 University of Chinese Academy of Sciences, Beijing 100049, China

**Keywords:** zero-bias Schottky diode, direct grounding, THz detector, radiometer receiver

## Abstract

Reliable terahertz radiometer receiver systems demand detectors that combine high sensitivity with structural robustness. This paper presents the design, fabrication, and characterization of a 380 GHz zero-bias detector based on an ACST Schottky diode. The detector uses a high-impedance grounding topology to reduce parasitic resonances and enhance output stability. A compact U-shaped waveguide transition is adopted to realize an inline port configuration. This configuration simplifies system integration. Measurements demonstrate a peak voltage responsivity of 2318 V/W and a linearity of 0.9996 at 380 GHz, validating the effectiveness of the proposed design. This work establishes a practical design approach for zero-bias terahertz detectors and supports future high-frequency radiometer and sensing applications.

## 1. Introduction

Occupying the spectral bridge between microwave electronics and infrared photonics, the terahertz (THz) regime has unlocked critical advancements in scientific and engineering domains [1]. To fully exploit the unique spectral characteristics and application potential of the THz band, the development of high-efficiency, practical receiver technologies is paramount. Among diverse architectures, direct detection receivers have garnered significant interest. By eliminating the complex local oscillator (LO) chain, these receivers provide compact size and low power consumption, which are particularly advantageous for spaceborne and airborne systems subject to stringent size, weight, and power (SWaP) constraints [2].

As the core component converting incident THz signals into measurable DC output, the detector plays a decisive role in determining overall sensitivity and linearity of the receiver [3]. Schottky diodes have benefited from decades of development in high-frequency electronics and remain a mature technology for extending electronic detection toward the THz regime [4]. Despite advancements in Schottky diode and micro/nano manufacturing technologies [5], designing zero-bias detectors operating above 300 GHz remains a formidable challenge. At THz frequencies, parasitic effects associated with the Schottky junction and surrounding metallization increasingly influence detector performance. Existing grounding strategies, such as direct wire bonding or conductive epoxy attachment at the probe tip, have demonstrated effectiveness across millimeter-wave and lower terahertz bands [6,7]. However, at higher frequencies, these methods tend to exhibit increased sensitivity to fabrication tolerances.

The 380 GHz atmospheric window, characterized by its low propagation loss and favorable dispersion, is of strategic importance [8]. Consequently, this frequency band has emerged as a prime candidate for demanding applications, ranging from 6G communications to deep-space exploration and radiometric remote sensing [9,10]. Detector designs specifically optimized for the 380 GHz atmospheric window while offering high sensitivity and compactness remain relatively limited. Representative state-of-the-art results include the waveguide-packaged zero-bias detectors from Virginia Diodes Inc. (VDI), which achieve responsivities of approximately 3000 V/W at 50 GHz and 100 V/W at 1100 GHz [11]. Within the sub-millimeter regime, the Institute of Microelectronics, Chinese Academy of Sciences, has reported a 270 GHz zero-bias detector based on an InP Schottky diode, achieving a typical responsivity of 1400 V/W across 260–280 GHz [12]. Chen et al. demonstrated zero-bias GaAs Schottky detectors at 89 GHz and 150 GHz with stable responsivity and excellent linearity [13]. These studies demonstrate the feasibility of THz zero-bias detection while also underscoring the importance of detector architectures.

This work presents a compact, fabrication tolerant zero-bias THz detector designed for operation in the 380 GHz atmospheric window. The detector utilizes a low barrier GaAs Schottky diode from ACST, enabling high sensitivity and room temperature operation without external bias circuitry. While the use of a commercial diode chip and conventional waveguide-to-planar transition concepts follows established practice, the originality of this work lies in a tailored co-design and integration strategy optimized for the 380 GHz band. A directly grounded high impedance transmission line topology, together with a hammer-head low pass filter (LPF), is adopted to suppress parasitic resonances and enhance high frequency stability. To improve system integrability, a compact U-shaped waveguide bend is incorporated to realize an inline port configuration. The design methodology provides a practical foundation for THz imaging and remote sensing receivers, establishing a transferable framework for future zero-bias detector implementations at even higher frequencies.

## 2. Circuit Design and Analysis

The operating principle of a zero-bias terahertz detector is based on the square-law rectification characteristics of the Schottky diode, which converts incident THz signal into a measurable dc output. The diode current–voltage relationship is expressed as [14]

(1) $$I\left(V\right)=I_{s}(e^{\frac{qV}{nkT}}-1)$$

where $I_{s}$ is the saturation current, q is the electron charge, V is the junction voltage, n is the ideality factor, k is the Boltzmann constant, and T is the absolute temperature.

Under small-signal excitation, a small amplitude terahertz voltage $\delta V=V_{p}\cos\left(\omega_{c}t\right)$ is superimposed on the DC bias $V_{0}$ (which is zero in this case). By expanding the diode current using a Taylor series around the operating point and neglecting higher-order terms, the current can be approximated as:

(2) $$i\left(V\right)\approx i\left(V_{0}\right)+V_{p}\cos\left(\omega_{c}t\right){\left.\frac{di}{dV}\right|}_{V_{0}}+\frac{V_{p}^{2}}{4}{\left.\frac{d^{2}i}{dV^{2}}\right|}_{V_{0}}+\frac{V_{p}^{2}\cos\left(2\omega_{c}t\right)}{4}{\left.\frac{d^{2}i}{dV^{2}}\right|}_{V_{0}}$$

The resulting signal comprises DC, fundamental, and second-harmonic components. The generated DC current is proportional to the square of the input voltage amplitude, enabling estimation of the RF input power through DC voltage measurement.

(3) $$I_{DC}\propto \delta V^{2}$$

**Figure 1:**
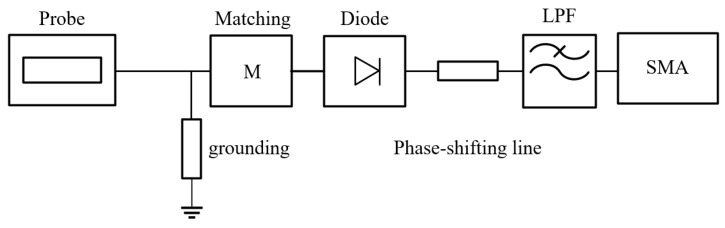
System topology of the 380 GHz zero-bias Schottky detector.

The system topology of the 380 GHz zero-bias detector is illustrated in Figure 1. The circuit consists of a waveguide-to-microstrip transition, an impedance matching network, a Schottky diode, a high-impedance grounding path, and an LPF. To streamline the circuit implementation, the conventional output matching section is replaced by a phase-shifting transmission line. This approach reduces circuit complexity and physical size while maintaining effective impedance matching.

As depicted in Figure 1, the incident THz signal is coupled into the microstrip line via a low loss waveguide-to-microstrip transition. The nonlinear Schottky junction performs the rectification, and the generated DC component is extracted by the LPF. At terahertz frequencies, the detector performance is determined not only by the intrinsic parameters of the Schottky diode but also by the electromagnetic coupling among circuit blocks, parasitic elements, and the physical layout. Optimization of the detector topology is essential for improving detection efficiency, enhancing high frequency stability, and robustness against fabrication tolerances.

### 2.1. Modelling of Schottky Diodes

Accurately predicting device behavior at 380 GHz requires a rigorous treatment of distributed parasitic effects, which render conventional lumped-element models insufficient. Consequently, a hybrid modeling strategy was adopted to characterize the quasi-vertical GaAs Schottky diode (ACST).

**Figure 2:**
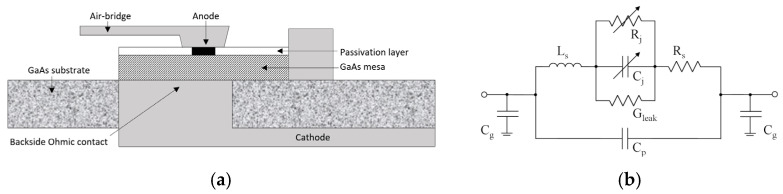
Structure and equivalent circuit of the Schottky diode: (**a**) cross-sectional schematic of the diode structure; (**b**) equivalent circuit model.

A full-wave electromagnetic analysis was first performed on the three-dimensional (3D) physical structure of the diode, including the GaAs mesa, air-bridge anode, and cathode pads, in order to extract frequency-dependent S-parameters. The cross-sectional schematic is illustrated in Figure 2a. These distributed parameters were then embedded into a nonlinear SPICE model in Figure 2b. This circuit includes the series resistance $R_{s}$, junction capacitance $C_{j}$, junction resistance $R_{j}$, series inductance $L_{s}$, shunt parasitic capacitance $C_{p}$, together with parasitic contributions from metallization, contacts, and fingers.

**Figure 3:**
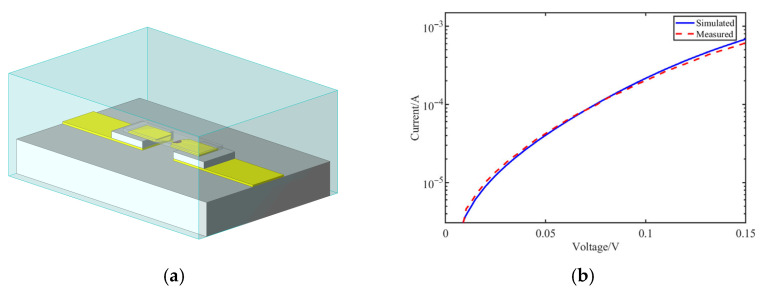
Schottky diode modeling and validation: (**a**) 3D electromagnetic model of the diode; (**b**) comparison between measured and simulated I–V characteristics.

Figure 3a shows the established 3D electromagnetic model of the diode. Key parameters, including the saturation current $I_{s}$, series resistance $R_{s}$, ideality factor n, and zero-bias junction capacitance $C_{j0}$, were extracted by fitting the model to the measured DC I-V characteristics. As shown in Figure 3b, the co-simulation results show good consistency with the measured data, validating the accuracy of the combined model for the subsequent detector design.

### 2.2. Waveguide-to-Microstrip Transition Structure

**Figure 4:**
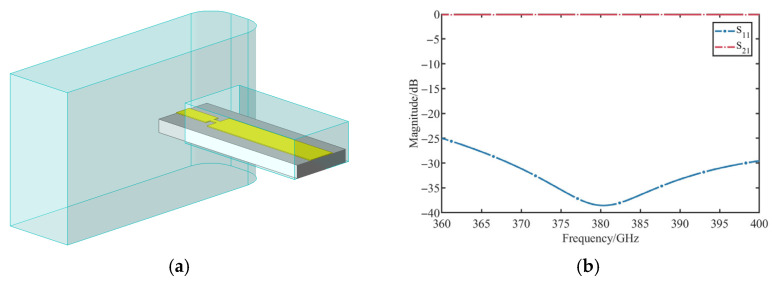
Waveguide microstrip transition Structure: (**a**) Geometry of the E-plane probe transition; (**b**) Simulated S-parameters of the transition.

Efficient energy coupling is critical for detector sensitivity. An E-plane probe transition was optimized for the 360–400 GHz band. Key geometric parameters, especially the probe insertion depth and backshort distance, were tuned by parametric sweeps. Figure 4a illustrates the optimized transition structure. As shown in the simulation results in Figure 4b, the transition achieves a return loss better than 25 dB and insertion loss below 0.15 dB across the target frequency band.

**Figure 5:**
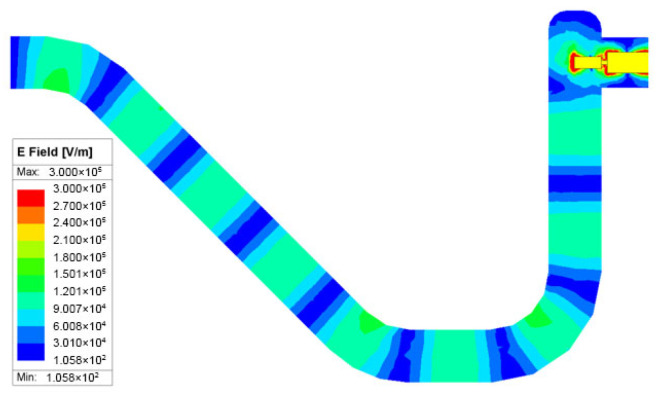
U-shaped waveguide bend structure.

However, standard E-plane probes dictate an orthogonal port arrangement, which complicates system stacking. To resolve this, a U-shaped waveguide bend was integrated to realize an inline port configuration, as shown in Figure 5. Unlike conventional mitered bends, which are prone to parasitic reflections and high-order mode excitation at terahertz frequencies, the proposed design features a large-radius continuous curvature. Field distribution simulations confirm that this smoothed profile ensures seamless energy transmission without significant radiation leakage or resonant accumulation, effectively satisfying the dual requirements of compactness and high-frequency signal integrity. The simulated insertion loss is less than 0.4 dB.

### 2.3. Grounding Design

In E-plane probe transitions, the DC return path is usually realized by extending the substrate across the waveguide aperture to contact with the cavity wall [15,16]. However, this method extends the substrate length, increasing mechanical fragility for thin quartz materials. Another grounding scheme relies on gold wire bonding [17,18]. At terahertz frequency, thinner gold wires are needed. However, the standard bonding processes typically utilize wires with diameters ranging from 12.5 µm to 25 µm, thinner gold wires pose challenges in terms of process stability and reliability [19].

**Figure 6:**
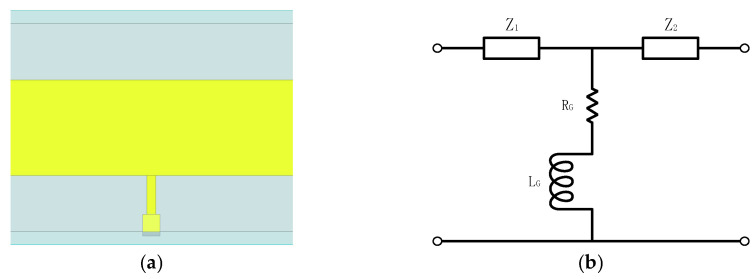
High-impedance direct-grounding structure: (**a**) physical layout of the grounding line; (**b**) equivalent circuit representation of the grounding path.

To mitigate the sensitivity of conventional grounding schemes to fabrication and assembly tolerances at terahertz frequencies, a high-impedance grounding path is adopted in this work. The grounding structure is shown in Figure 6a. Placing the grounding line upstream of the input matching network facilitates a more uniform distribution of grounding points. This configuration reduces the sensitivity of the grounding impedance to position variations, improving robustness against fabrication and assembly tolerances. The grounding wire behaves as a distributed element whose high-frequency response can be approximated by a series inductance and resistance. The equivalent circuit of the grounding path is shown in Figure 6b.

The geometrical dimensions of the grounding wire can be precisely controlled during fabrication, and its high frequency behavior can be approximated by a series inductance $L_{G}$ and resistance $R_{G}$, expressed as:

(4) $$L_{G}=\mu_{0}\cdot \frac{l}{2\pi }\cdot \left(\ln\left(\frac{4l}{d}\right)+\frac{\mu_{r}\tanh\left(\frac{4d_{s}}{d}\right)}{4}-1\right)$$

(5) $$R_{G}=\left\{\begin{array}{c} \left(\frac{4\rho l}{\pi d^{2}}\right)\cdot \cosh\left[0.041{\left(\frac{d}{d_{s}}\right)}^{2}\right],\;\;\frac{d}{d_{s}}\leq 3.394\\ \left(\frac{4\rho l}{\pi d^{2}}\right)\cdot \left(\frac{0.25d}{d_{s}}+0.2654\right),\;\;\frac{d}{d_{s}}>3.394 \end{array}\right.$$

where $\mu_{0}$ is the permeability of free space, l is the wire length, d is the wire diameter, $d_{s}$ is the skin depth at the operating frequency, and ρ denotes the electrical resistivity.

### 2.4. Low Pass Filter Design

**Figure 7:**
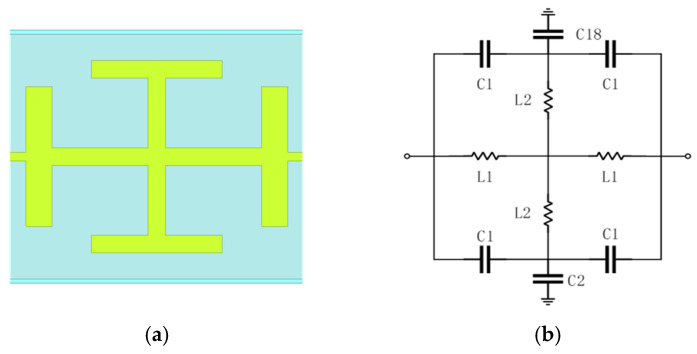
Hammer-head low-pass filter unit cell: (**a**) physical layout of the microstrip structure; (**b**) equivalent circuit.

To extract the rectified DC signal while rejecting the terahertz carrier, a compact filter with steep roll-off is required. Traditional stepped-impedance filters are often too lengthy for integrated terahertz modules [20]. To achieve steep stopband rejection within a compact footprint, a Hammer-head topology [21] is adopted. As shown in Figure 7a, this structure introduces capacitive loading via a planar expansion element on the microstrip line, which effectively sharpens the cutoff response and enhances high-frequency attenuation without substantially increasing the circuit length. The equivalent circuit model of the cell structure is shown in Figure 7b.

**Figure 8:**
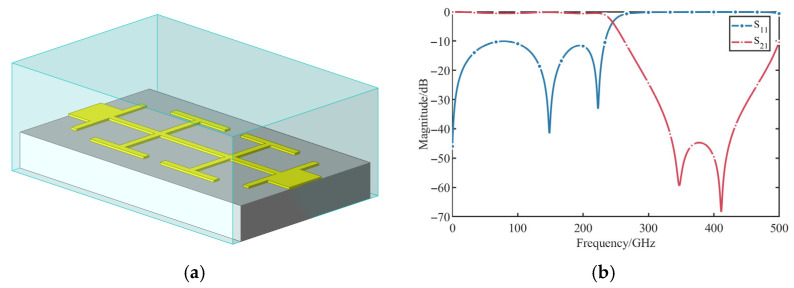
Cascaded hammerhead LPF: (**a**) 3D model of the LPF; (**b**) Simulated S-parameters of the LPF.

To enhance the stopband attenuation, two Hammer-head cells are cascaded. The resulting dual-stage LPF structure and its simulated frequency response are shown in Figure 8. The simulation results indicate an attenuation level exceeding 42 dB across the 340–420 GHz bandwidth, while maintaining low insertion loss at low frequency, ensuring that the DC signal generated by the detector can pass through the filter smoothly. The proposed LPF simultaneously offers structural compactness and superior stopband performance.

### 2.5. Overall Simulation and Analysis

**Figure 9:**
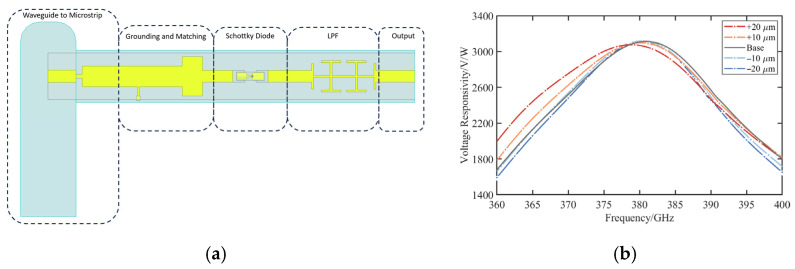
Simulation results of the 380 GHz zero-bias detector: (**a**) Integrated circuit model; (**b**) Voltage responsivity for different grounding points.

The individual sub-circuits were integrated into a system-level model for final optimization, as shown in Figure 9a. The lengths of the phase-shifting line and the diode input transmission lines were adjusted to compensate for the imaginary part of the input impedance of the diode-LPF cascade at 380 GHz. Considering the compactness requirements of the detector circuit, a single stub matching network is adopted to realize high responsivity detection over the operating bandwidth.

To validate the manufacturing robustness, a tolerance-aware parametric analysis was conducted by introducing controlled perturbations to the grounding position. Grounding offsets of ±10 μm and ±20 μm are incorporated into the co-simulation model, and the corresponding results are presented in Figure 9b. Simulation results show that the detector achieves a peak voltage responsivity of about 3000 V/W near 380 GHz and maintains stable performance within the range of ±20 μm offset, with only a slight responsivity variation and no observable frequency offset. These results demonstrate that the proposed grounding topology effectively mitigates performance variations caused by fabrication and provides robust support for stable and reproducible high sensitivity detector performance.

## 3. Fabrication and Measurements

**Figure 10:**
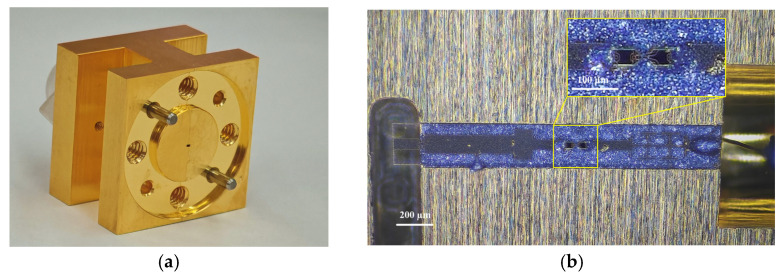
Fabricated 380 GHz zero-bias detector: (**a**) detector module; (**b**) Optical image of the detector circuit.

The detector is implemented in a split-block waveguide architecture fabricated from gold-plated copper, ensuring high conductivity and minimizing surface losses. As shown in Figure 10a, the input port adopts standard WR-2.2 waveguide interface, and the volume of the whole detection module is 20 × 20 × 15 mm^3^. The detector circuit was fabricated on a quartz substrate with a relative permittivity of 3.78 and a thickness of 50 μm. As shown in Figure 10b, the substrate is mounted into the split-block channel using conductive silver epoxy, and direct grounding is established. The DC output port was connected to an SMA probe via gold wire bonding. During this practical assembly process, the manual dispensing of conductive silver epoxy and the placement of the quartz substrate inherently introduce microscopic alignment variations, typically within the range of 10 to 20 μm.

**Figure 11:**
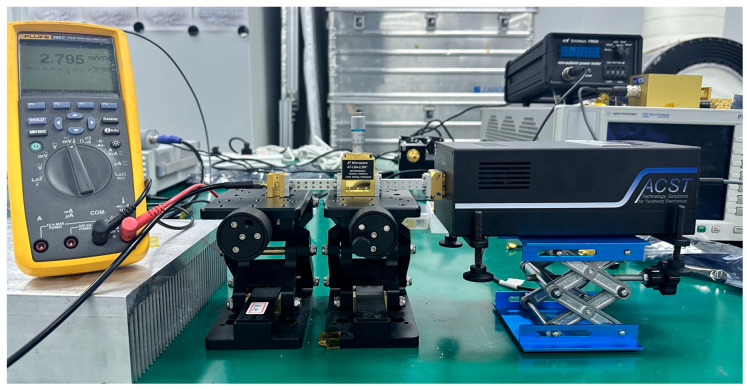
Testing environment.

The experimental setup is shown in Figure 11. A PSG Analog Signal Generator was used as the small-signal source, and the terahertz signal was generated through a frequency-multiplier chain provided by ACST. The signal is then delivered to the detector under test via a variable attenuator. To characterize the detector voltage responsivity, the input power at the detector was calibrated using a VDI PM5B power meter (Virginia Diodes Inc., Charlottesville, VA, USA), and the input level was maintained at approximately −30 dBm. For the linearity measurement, the carrier frequency is kept constant while the input power is swept by adjusting the attenuator, and the resulting DC voltage response is recorded using a FLUKE digital multimeter(Fluke Corporation, Everett, WA, USA).

**Figure 12:**
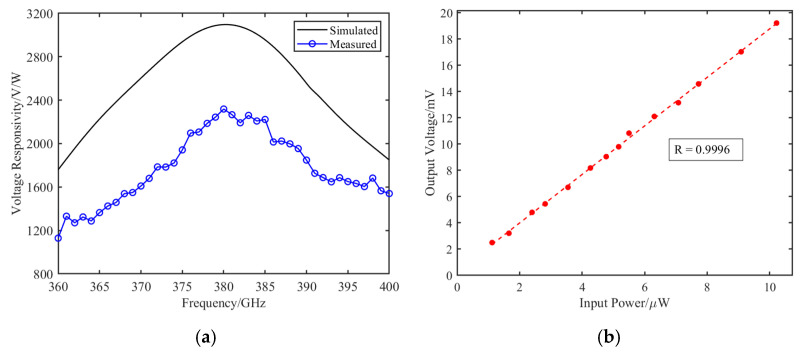
Measured performance: (**a**) voltage responsivity; (**b**) linearity.

The relationship between the measured voltage responsivity and frequency is shown in Figure 12a. Within the frequency range of 360–400 GHz, the detector achieves a peak voltage responsivity of 2318 V/W at 380 GHz, demonstrating that the zero-bias Schottky detector exhibits favorable detection performance in this frequency band. For a zero-bias Schottky diode, the total noise is dominated by thermal noise. Using the typical junction resistance $R_{j}$ of 2500 Ω at room temperature (T = 290 K), the theoretical thermal noise voltage $V_{n}=\sqrt{4k_{B}TR_{j}}$ is calculated to be approximately 6.32 nV/√Hz. Given the measured peak voltage responsivity of 2318 V/W at 380 GHz, the theoretical noise limited equivalent power (NEP) is estimated to be roughly 2.73 pW/√Hz.

Linearity, a critical metric for accurate radiometric brightness temperature retrieval, was evaluated by sweeping the input power at 380 GHz. As shown in Figure 12b, the detector exhibits a clear linear response over the measured input power range.

Compared with the simulated results, the measured responsivity exhibits a certain degree of degradation across the entire frequency band. This discrepancy is primarily attributed to several practical non-idealities at sub-millimeter wavelengths. First, the nonlinear diode model employs nominal parameters, whereas the effective series resistance and junction capacitance of the assembled Schottky diode can deviate from the modeled values under practical DC and high frequency operating conditions, which directly affects the RF-to-DC conversion efficiency and the peak responsivity. Furthermore, parasitic effects introduced by the conductive silver epoxy assembly, microscopic substrate placement inaccuracies, and the output gold wire bonding all collectively contribute to the overall responsivity degradation. Despite these differences, the measured results are consistent with the simulation in terms of the overall trend and the location of the peak responsivity, which verifies the validity of the proposed design approach. Table 1 presents a comparison of the voltage responsivity of the proposed detector with other reported terahertz detectors.

The results demonstrate that the detector is specifically optimized for the 380 GHz atmospheric window, achieving a highly competitive peak voltage responsivity of 2318 V/W compared to the commercial VDI WR2.2 detector. The proposed design achieves competitive detection performance within a comparable frequency range.

## 4. Conclusions

A 380 GHz zero-bias Schottky diode waveguide detector is demonstrated, featuring an integrated inline U-shaped waveguide interface, a broadband waveguide-to-microstrip transition, and a high impedance direct grounding topology. The proposed grounding strategy significantly reduces sensitivity to assembly misalignment. Simulations indicate that a ±20 μm lateral grounding offset causes only a minor degradation in peak responsivity and no pronounced center frequency shift, which is beneficial for repeatable manufacturing. A fabricated split-block prototype was characterized from 360 to 400 GHz, achieving a measured peak voltage responsivity of 2318 V/W at 380 GHz, and maintains linearity of 0.9996. These results validate that the proposed transition and grounding co-design enables compact, tolerance resilient THz detector modules suitable for radiometer receivers.

## Figures and Tables

**Table 1:** Comparison with related work.

Ref.	Frequency	Voltage Responsivity	Technology
[11]	330–500 GHz	Typ: 1250 V/W	VDI WR2.2 ZBD
[13]	85–95 GHz 145–155 GHz	Typ: 2500 V/W (85–95 GHz) Typ: 1600 V/W (145–155 GHz)	ACST SBD
[17]	140–165 GHz	1211 V/W (Max @147 GHz)	Schottky diodes
[18]	210–220 GHz	Typ: 1800 V/W	VDI G-band SBD
[22]	0.1–0.8 THz	320 V/W (@300 GHz)	InGaAs/InP SBD
[23]	330–500 GHz	2123 V/W (Max @340 GHz)	InP TB-RTD
This work	360–400 GHz	2318 V/W (Max @380 GHz)	ACST SBD

## Data Availability

The data that support the findings of this study are available from the corresponding author upon reasonable request.
